# Increasing Terbinafine Resistance in Danish *Trichophyton* Isolates 2019–2020

**DOI:** 10.3390/jof8020150

**Published:** 2022-01-31

**Authors:** Karen Marie Thyssen Astvad, Rasmus Krøger Hare, Karin Meinike Jørgensen, Ditte Marie Lindhardt Saunte, Philip Kjettinge Thomsen, Maiken Cavling Arendrup

**Affiliations:** 1 Unit of Mycology, Statens Serum Institut, DK-2300 Copenhagen, Denmark; rmj@ssi.dk (R.K.H.); kmj@ssi.dk (K.M.J.); disa@regionsjaelland.dk (D.M.L.S.); maca@ssi.dk (M.C.A.); 2 Department of Dermatology, Zealand University Hospital, DK-4000 Roskilde, Denmark; 3 Department of Clinical Medicine, Faculty of Health Science, University of Copenhagen, DK-2100 Copenhagen, Denmark; 4 Department of Clinical Microbiology, Aalborg University Hospital, DK-9220 Aalborg, Denmark; p.thomsen@rn.dk; 5 Department of Clinical Microbiology, Rigshospitalet, DK-2100 Copenhagen, Denmark

**Keywords:** *Trichophyton rubrum*, *Trichophyton interdigitale*, *Trichophtyon mentagrophytes*, *Trichophyton indotineae*, squalene epoxidase, SQLE, terbinafine, *Trichophyton benhamiae*, dermatophyte resistance

## Abstract

Terbinafine resistance in *Trichophyton* species has emerged and appears to be increasing. A new EUCAST susceptibility testing method and tentative ECOFFs were recently proposed for *Trichophyton*. Terbinafine resistance and target gene mutations were detected in 16 Danish isolates in 2013–2018. In this study, samples/isolates submitted for dermatophyte susceptibility testing 2019–2020 were examined. Species identification (ITS sequencing for *T. mentagrophytes/T. interdigitale* species complex (SC) isolates), EUCAST MICs and squalene epoxidase (SQLE) profiles were obtained. Sixty-three isolates from 59 patients were included. *T. rubrum* accounted for 81% and *T. mentagrophytes/T. interdigitale* SC for 19%. Approximately 60% of *T. rubrum* and *T. mentagrophytes/interdigitale* SC isolates were terbinafine non-wildtype and/or had known/novel SQLE mutations with possible implications for terbinafine MICs. All infections with terbinafine-resistant *T. mentagrophytes/interdigitale* SC isolates were caused by *Trichophyton indotineae*. Compared to 2013–2018, the number of patients with terbinafine-resistant *Trichophyton* isolates increased. For *T. rubrum*, this is partly explained by an increase in number of requests for susceptibility testing. Terbinafine-resistant *T. indotineae* was first detected in 2018, but accounted for 19% of resistance (4 of 21 patients) in 2020. In conclusion, terbinafine resistance is an emerging problem in Denmark. Population based studies are warranted and susceptibility testing is highly relevant in non-responding cases.

## 1. Introduction

Dermatophytosis (syn. tinea) is the most common fungal infection of keratinised tissue (hair, nail and skin). Taxonomic revision in 2017 led to 9 clades of *Arthrodermataceae* species being recognised (*Trichophyton, Epidermophyton, Nannizia, Paraphyton, Lophophyton, Micropsporum, Arthroderma, Ctenomyces* and *Guarromyces*) [1]. Furthermore, it was recognised that the *Trichophyton mentagrophytes/interdigitale* species complex (SC) was composed of both the anthropophilic species *T. interdigitale* and the predominantly zoophilic species *T. mentagrophytes*. In 2020, *Trichophyton indotineae* was proposed as a new species (formerly *T. mentagrophytes* ITS genotype VIII) after having first emerged as a cause of recalcitrant tinea corporis and tinea cruris in India [2,3].

Terbinafine is a first-line agent for *Trichophyton* infections, whether topical or systemic depends on severity and hair/nail involvement [4]. Reports of terbinafine resistance were almost absent until 2017 [5]. Terbinafine resistance is mainly coupled to various mutations in the terbinafine target gene squalene epoxidase (SQLE). It has sporadically been reported in isolates of *T. rubrum* and *T. interdigitale,* especially from Europe [6,7,8,9,10]. In India and other Asian countries, resistance has emerged as a clonal outbreak of terbinafine-resistant *T. indotineae* [11,12,13]. The cause of this outbreak has been speculated to be driven by the uncontrolled over-the-counter sale of topical cream containing both steroids, antifungals and antibiotics [14]. *T. indotineae* has subsequently been reported from several other countries including Japan, Cambodia, Iran, Bahrain, Switzerland, Greece, Finland, and Germany and is thought introduced by inhabitants or visitors from the Indian subcontinent [2,15,16,17,18,19,20,21]. As dermatophytes are slow growing, PCR is increasingly applied for diagnostics, but current methods are unable to differentiate between *T. mentagrophytes/T. interdigitale* complex species. Thus, correct species ID normally relies on DNA sequencing [3,21]. Identification of these isolates has caused taxonomical confusion, as many isolates, including those in the literature and databases, have been labelled as *T. interdigitale* or *T. mentagrophytes* [22]. Previously published terbinafine-resistant *T. interdigitale* or *T. mentagrophytes* isolates from various other countries may also be *T. indotineae*, and updated data on epidemiology is warranted [23,24,25].

Resistance rates depend on the method used and how the MICs are interpreted in the absence of formal breakpoints. In 2020, EUCAST established a reference method E.Def. 11.0 for testing microconidia forming dermatophytes, adopting a 50% endpoint using a spectrophotometer, addition of cycloheximide end chloramphenicol to the media and 4–7 days incubation time [26]. Tentative ECOFFs for terbinafine, amorolfine, itraconazole and voriconazole were proposed for *T. rubrum* and *T. indotineae*, based on a multicenter study [27,28].

In Denmark, surveillance programs are established for candidaemia and azole resistant *Aspergillus fumigatus*, but not for dermatophytosis. In 2019, a retrospective laboratory study demonstrated 14 cases of terbinafine-resistant *Trichophyton* isolates, with all isolates harbouring SQLE mutations. During the last two years, more specimens and isolates have been referred for susceptibility testing. We report susceptibility data and SQLE profiles for these isolates and compare with previous years. Moreover, all isolates of the *T. mentagrophytes/T. interdigitale* SC were ITS sequenced to ensure correct species identification. Part of these data were presented as a Mini Oral Flash Session at ECCMID 2021 (#2185).

## 2. Materials and Methods

### 2.1. Inclusion Criteria and Methods for Culture and Identification

Until December 2020, dermatophyte susceptibility testing in Denmark was centralised at Statens Serum Institut. Inclusion criteria were non-duplicate microconida-forming dermatophyte isolates cultured from clinical samples or submitted as pure cultures and for which identification and susceptibility testing during 2019–2020 was requested. Duplicate isolates were defined as identical isolates with the same susceptibility profile and isolated ≤60 days apart (*n* = 4). Sequential isolates after 60 days were included as reinfection could not be excluded and to allow comparison between years prospectively (*n* = 4; three in 2019, one in 2020, and separated by 70–231 days (mean 161 days)). In total, sixty-three non-duplicate isolates were included from 59 patients. Resistance rates were determined at patient level. Culturing was performed on Sabouraud glucose agar supplemented with chloramphenicol and cycloheximide (SSI Diagnostica, Hillerød, Denmark), and cultures were incubated at 25 °C for as long as 4 weeks. Identification to genus and species level was performed by micro- and macro morphology and ITS sequencing when needed. ITS sequencing was performed or re-evaluated in 2021 for all isolates of the *T. mentagrophytes/T. interdigitale* SC as previously described [6]. DNA was extracted by obtaining fungal material from >5 day cultured plates and subjected to the automated NucliSENS easyMag platform (bioMérieux Nordic, Gothenburg, Sweden) and eluted in 100 µL. ITS PCR was performed using 2 µL DNA in a 25 µL reaction mix containing 0.25 µM universal primers ITS5 (5′-GGAAGTAAAAGTCGTAACAAGC-3′) and ITS4 (5′-TCCTCCGCTTATTGATAGC-3′) and ×1 Extract-N-Amp PCR ReadyMix (Sigma-Aldrich Chemie GmbH, Schnelldorf, Germany) in a ×35 cycle program with annealing temperature of 57 °C. PCR amplicons were subjected for purification and Sanger sequencing at Macrogen, Holland. DNA sequences were analysed and assembled using CLC Main Workbench v20 and v21 (Qiagen, Aarhus, Denmark). Species identification was based on a sequence similarity of 100% using well-defined reference strains described by Tang et al. [3] ATCC 9533 (Genbank ID KJ606115) for *T. interdigitale*, ATCC 16781 (=CBS 623.66, Genbank ID KJ606079) for *T. benhamiae* and NUBS19006 (Genbank ID LC508024) for *Trichophyton indotineae* (Kano et al. [2]). ITS sequences were 100% identical on species level and a representative sequence for each species was submitted to Genbank (Accession, OM281733, OM281734, OM281735 and OM281736). None of the included Danish isolates were identified as *T. mentagrophytes* genotypes. Subsequently, species reclassification was also performed for the terbinafine-resistant *T. interdigitale* isolates included in a previous study from 2013–2018 [6]. The number of patients with terbinafine non-susceptible isolates (either resistant or with SQLE mutations suspected to confer increased terbinafine MICs) was compared between the present study and the aforementioned 2013–2018 study period [6].

### 2.2. Antifungal Susceptibility Testing

Microtiter plates (cell culture-treated Thermo Fisher Scientific (Nunc) MicroWell 96-well microplates, catalog no. 167008; Sigma-Aldrich, Brøndby, Denmark) were prepared according to the EUCAST reference method E.Def 11.0 using double-concentrated RPMI 1640 buffered with 3-(N-morpholino) propanesulfonic acid (MOPS) and supplemented with 2% glucose (SSI Diagnostica, Hillerød, Denmark) and 1% dimethyl sulfoxide (DMSO) (Sigma-Aldrich) [26], and adopting serial 2-fold dilution with pipette tip changes in columns 4 and 7 [29]. Plates were frozen at −80°C for at least 24 h before use. Stock solutions of antifungal compounds were prepared in DMSO (5000 mg/L; Sigma-Aldrich). The antifungals (manufacturer; concentration ranges) applied were as follows: terbinafine and posaconazole (Sigma-Aldrich, Brøndby, Denmark and Merck, NJ, USA; 0.004 to 4 mg/L), itraconazole (Sigma-Aldrich; 0.016–16 mg/L and 0.004–4 mg/L), voriconazole (Pfizer A/S, Ballerup, Denmark until Nov. 2019 followed by Sigma-Aldrich, Brøndby, Denmark; 0.016–16 mg/L and 0.004–4 mg/L), isavuconazole (Basilea, Basel, Switzerland; 0.016–16 mg/L and 0.008–8 mg/L) and olorofim (F2G, Manchester, UK; 0.001–1 mg/L). Inoculum suspensions were prepared according the EUCAST E.Def 11.0 method in sterile water supplemented with 0.1% Tween-20 (Sigma-Aldrich), filtered through a sterile filter with a pore diameter of 11 µm (Millipore Nylon Net Filter 11µm NY11, Merck Millipore Ltd., Tullagreen, Carrigtohill, County Cork, Ireland) to remove hyphae and diluted 1:10 with sterile distilled water to obtain a final working inoculum of 2–5 × 10^5^ cfu/mL [26]. *C. krusei* ATCC6258, *C. parapsilosis* ATCC22019, *A. fumigatus* ATCC204305 and *A. flavus* ATCC204304 were used as quality controls for susceptibility testing [30] and were read after 1 day (yeast; 50% inhibition endpoint) or 2 days (moulds, visual no-growth endpoint) of incubation at 37 °C. For the *Trichophyton* isolates, cycloheximide and chloramphenicol were added to the inoculum solution as per protocol (final concentrations in the inoculated susceptibility plate, 50 mg/L and 300 mg/L, respectively). Plates were read using a 50% inhibition endpoint compared to antifungal free control wells, using a spectrophotometer (490 nm wavelength). Incubation time at 25–28 °C was 5(–7) days (preferentially 5 days) [26]. Prior to the publication of the EUCAST reference method 11.0 for dermatophytes in April 2020, MICs had been determined visually. For these isolates, stored files with spectrophotometer data were reanalysed, using the established reference 50% endpoint criterion. Thus, all presented MICs in this study are generated according to the E.Def 11.0. The EUCAST tentative ECOFFs (tECOFFs) for terbinafine, itraconazole and voriconazole were used to determine the non-wildtype (NWT) proportion of *T. rubrum* and *T. indotineae* isolates. Isolates of *T. interdigitale* were considered WT for terbinafine, voriconazole and itraconazole if: (1) MICs were below the tECOFFs for the closely related species *T. indotineae* and (2) a unimodal MIC distributions was found [26,28].

### 2.3. SQLE Sequencing

SQLE sequencing of *T. rubrum* and *T. mentagrophytes/interdigitale* SC (including *T. benhamiae*) were done as previously described [6]. The entire gene encoding squalene epoxidase was amplified using the same reaction conditions as for ITS but with the primers; TRUB SE-F0 (5′-TTACCCCATCAATAAGTTACTAC-3′) and TRUB SE-R0 (5′-GAGTTAGAGATAAGCCTATCTGC-3′) for *T. rubrum* (annealing temperature 54 °C) and Tricho SE-F0 (5′-TGACAGCGACAAGTGCCA-3′) and TINT SE-R0 (5′-AAAGAGCTAGAGATAAGCCTATCTG-3′) for *T. interdigitale, T. indotineae* and *T. benhamiae* (annealing at 57 °C). PCR products were purified and Sanger sequenced at Macrogen, Netherlands using additional sequencing primers; TRUB SE-F2 (5′-AATATCTCCCCATACAACCAG-3′) and TRUB SE-R2 (5′-AACCC-TCCCTTCTCCAACGCA-3′) for *T. rubrum* and TRI SE-F3 (5′-GGAATATCTCCCCATACAACCAG-3′) and TRI SE-R3 (5′-CCTCCCTTCTCC-AACGCAG-3′) for the non-*rubrum* species. Sequences were assembled to wild-type reference sequences for *T. rubrum* (Genbank ID NW_003456423, locus TERG_05717), *T. interdigitale* (Genbank ID KK204440 locus EZF33561), *T. indotineae* (Genbank ID MW187977) and *T. benhamiae* (Genbank ID NW_003315110, locus ARB_06092). The *T. indotineae* reference sequence differs 6 bp, 66 bp and 88 bp from the reference sequences of *T. interdigitale*, *T. rubrum* and *T. benhamiae,* respectively, and thus allow further support of the species identification obtained by ITS. Genbank accession numbers corresponding to each squalene epoxidase profile for the four *Trichophyton* species are OM313296-OM313313.

## 3. Results

### 3.1. Isolates and Identification

Sixty-three *Trichophyton* isolates from 59 patients were included. The number of isolates increased from 19 isolates (16 patients) in 2019 to 44 isolates (43 patients) in 2020. *T. rubrum* was found in 81% of patients and *T. mentagrophytes/T. interdigitale SC* in 19% (Table 1). Upon ITS and SQLE sequencing, 7 of 11 *T. interdigitale* isolates were reclassified as *T. indotineae*, which had not been identified in Denmark previously. On retrospective analysis of ITS and SQLE data from the two previously reported terbinafine-resistant *T. interdigitale* isolates, one with an F397L substitution (2018) was reclassified as *T. indotineae*, making this the earliest resistant *T. indotineae* isolate found in Denmark [6].

### 3.2. Antifungal Susceptibility Testing

**Figure 1:**
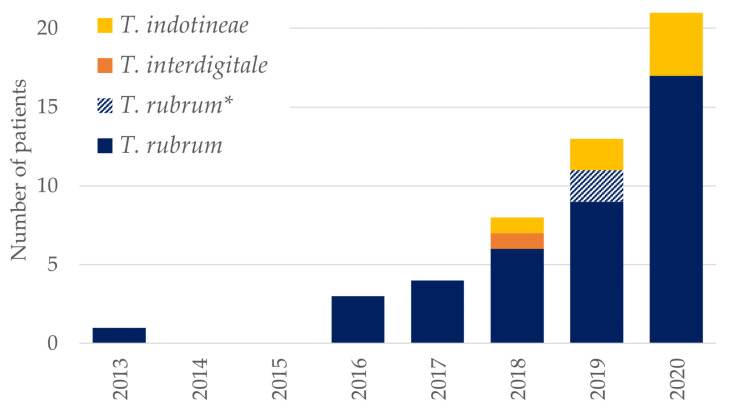
Annual number of Danish patients with terbinafine-resistant *Trichophyton* isolates or *Trichophyton* isolates with SQLE mutations associated with terbinafine resistance. (*) Two *T. rubrum* patients in 2019 were also included in the previous study (isolates same ID and SQLE profile found in 2017 and 2018, respectively) and are indicated in stripes [6].

MIC determination was possible for terbinafine for 43/51 isolates of *T. rubrum* (two were not susceptibility tested, one had bacterial overgrowth and five had insufficient growth to allow MIC determination). If comparing the number of patients with resistant isolates (based on phenotypic resistance and/or detection of SQLE mutations associated with resistance) over time to a previous study [6], there was a clear increase over time, even if excluding repeatedly culture positive isolates from four individual patients (Figure 1).

The terbinafine MIC distribution for *T. rubrum* was trimodal and with 55.8% of isolates being NWT (Table 2). For *T. indotineae*, all seven isolates were terbinafine NWT with MICs ≥ 2 mg/L whereas we found low MICs for *T. interdigitale* and *T. benhamiae* isolates (Table 2). One isolate of *T. rubrum* was NWT for both voriconazole and itraconazole, with two additional isolates being NWT for only one of the azoles, leading to NWT rates of 4.5% for both drugs. Low azole MICs were detected for the remaining *Trichophyton* species. Low olorofim MICs were found for all *Trichophyton* isolates (Table 2).

### 3.3. SQLE sequencing

**Figure 2:**
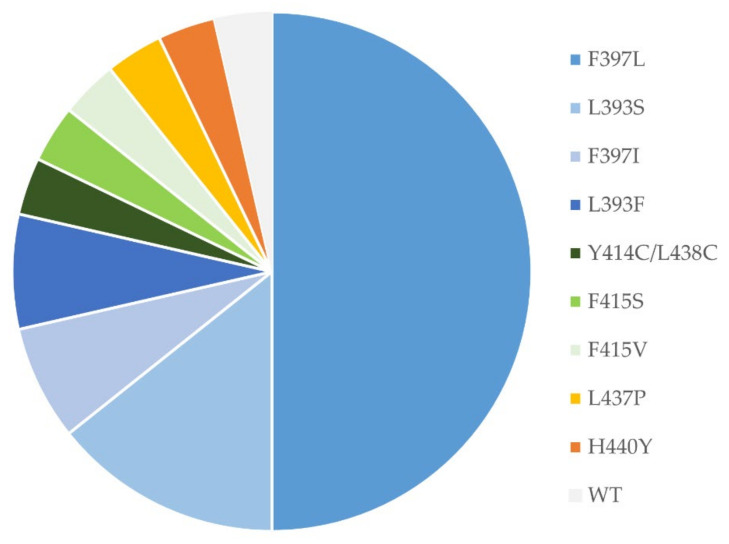
SQLE sequences for *T. rubrum* isolates from the patients (*n* = 28) with terbinafine-resistant isolates and/or SQLE profiles considered potentially significant for terbinafine susceptibility 2019–2020.

All *T. interdigitale* and *T. benhamiae* isolates were wild-type (WT), whereas 31 of 51 (61%) isolates of *T. rubrum* and 7 of 7 (100%) of *T. indotineae* isolates had missense mutations (Table 3). The most commonly found mutation led to the F397L substitution for both species, followed by substitutions of L393 (Table 3). For *T. rubrum*, additional previously described mutations conferring elevated terbinafine MICs were discovered. One isolate harboured two not previously published amino acid changes Y414C/L438C, adjacent to F415 and H440, respectively. Finally, an I479V amino acid substitution was discovered in a susceptible isolate (Figure 2, Table 3). Only three mutations were detected for the six patients with *T. indotineae* (Table 3).

## 4. Discussion

Detection and identification of dermatophytes are routinely performed locally at several departments of clinical microbiology in Denmark and only selected isolates are referred for further susceptibility testing. Yet, the proportion of *T. rubrum* in this article reflects that *T. rubrum* was the most prevalent (~80%) species in Denmark, in agreement with an epidemiological study from 2003 [31]. Approximately 17% of the isolates belonged to the *T. interdigitale/T. mentagrophyte*s SC, with terbinafine-resistant *T. indotineae* appearing for the first time in Denmark in a sample from 2018.

Adopting the EUCAST tECOFFs all *T. interdigitale* and *T. indotineae* isolates were azole WT, whereas three *T. rubrum* isolates were classified as NWT to itraconazole or voriconazole or both. These findings suggest a low frequency of azole resistance among Danish *Trichophyton* isolates in general, which potentially reflects the Danish guideline recommendation to prefer terbinafine over azoles as first line agent to avoid a collateral selection pressure on the *Candida* flora [4]. In India, varying levels of itraconazole resistance in *T. mentagrophytes/T. interdigitale* complex has been reported using the CLSI method (e.g., 0.2% to approx. 25% (among terbinafine resistant isolates)) [11,13,32]. To what extent this discrepancy between Europe and India reflects technical issues including different susceptibility testing methodologies and criteria for MIC interpretation, or reflects true differences in antifungal susceptibility remain unclear. For itraconazole, trailing growth may lead to wide MIC distributions and impact the observed resistance rate particularly when the MIC is determined using a stringent endpoint (complete or 90% inhibition) as adopted by CLSI in comparison with the 50% endpoint adopted by EUCAST [27]. However, India is also known for a high over-the-counter use of antifungal containing medication and a true higher resistance rate is therefore not unexpected. Of note, a number of Indian isolates that harbour the A448T mutations in the SQLE gene has been reported, which has been hypothesized to elevate azole MICs in conjunction with other mutations, but other resistance mechanisms such as target gene mutations or upregulated efflux pumps may also be involved [13,33,34,35,36]. The in vitro activity of isavuconazole was comparable to that of voriconazole on a mg/L basis. Given that voriconazole is associated with phototoxicity and skin cancer, this finding suggest that a potential future role as an alternative to voriconazole in difficult to treat cases might warrant further investigation. This study also confirmed a potent in vitro activity of olorofim. The mode of action of olorofim is different from that of licensed agents. Thus, this agent may also deserve investigation for terbinafine and/or azole resistant dermatophytosis.

For terbinafine, the EUCAST tECOFFs successfully separated isolates with and without SQLE resistance mutations (Table 3). Terbinafine resistance rates up to 59–81% have been reported in *T. indotineae* from India, even though noticeable regional differences are observed [13,33,37]. All of our *T. indotineae* isolates were NWT but none of our *T. interdigitale* when categorized as such adopting the tECOFFs for *T. indotineae*. It remains to be seen, however, if WT MICs and future species specific ECOFFs for the species within this species complex may differ [27,28]. One SQLE WT *T. rubrum* isolate had a terbinafine MIC of 0.06 mg/L, which is one dilution step above the tECOFF. This could be due to biological variation in susceptibility testing or alternative mechanisms of resistance. Various efflux pumps have also been described to cause terbinafine resistance [38,39]. Another *T. rubrum* isolate with an H440Y alteration had a WT MIC of 0.03 mg/L (day five). MIC testing was repeated twice and MICs were 0.06–0.125 mg/L if read on day 6 instead of day 5, which would be considered NWT. This suggests that H440Y may confer slightly elevated MIC levels leading to random susceptibility classification with unknown clinical importance [6,9].

SQLE profiling showed that the majority of terbinafine resistance in Danish isolates was caused by F397L, leading to high terbinafine MICs (0.5–>4 mg/L). This was followed by L393F and L393S, which are also acknowledged causes of resistance in various countries, mainly in Asia and Europe [19]. One *T. rubrum* isolate with an L437P substitution had a terbinafine MIC of 1 mg/L, suggesting that this novel alteration may be of clinical relevance although this remains to be confirmed. Three *T. rubrum* isolates grew insufficiently for susceptibility testing and harboured SQLE substitutions (F415S, F415V and Y414C/L438C) (Table 3). Alterations in F415 and/or H440Y, H440Y/F484Y and I121M/V237I have previously been reported in *T. rubrum* or H440Y in *T. indotineae* isolates with slightly elevated terbinafine MICs from Switzerland, Denmark or India [6,9,33]. Substitutions in nearby codons, such as Y414C and L438C, may also be significant for terbinafine susceptibility, although MICs may be close to the tECOFF [6,9]. Moreover, alteration Q408L or Q408L/A448T in *T. mentagrophytes* has been associated with elevated MICs in isolates from Switzerland and India [9,13,18], whereas L335F/A448T and S395P/A448T were associated with discreet MIC elevations in *T. mentagrophytes* isolates from India [13]. In agreement with our findings, prior reports found that *T. rubrum* isolates with F415S and in our study, also F415V variants have more retarded growth than WT isolates [9]. This would indicate that isolates with some mutations may be challenging to susceptibility test and additional information can be obtained through SQLE sequencing. In contrast to the alterations discussed above, some alterations are found in both susceptible and resistant isolates or exclusively in susceptible isolates, suggesting they do not affect the terbinafine susceptibility. This was the case for the I479V alteration found in an isolate with an MIC of 0.016 mg/L and thus identical to the modal MIC of the WT population. Similarly, the S443P alterations in *T. mentagrophytes* has been found in both resistant and susceptible isolates from India [13]; single A448T alternations almost exclusively in susceptible isolates of *T. mentagrophytes* or *T. indotineae* from Germany, Iran, and India [13,21,33,40]; and V444I/A448T and K276N/L419F alterations in *T. mentagrophytes* isolates from Germany or China were not associated with MIC elevations [21,33].

We found a high rate of terbinafine resistance in submitted *T. rubrum* isolates of approx. 56% based on susceptibility testing and 61% if including isolates with potentially significant SQLE mutations but insufficient growth for susceptibility testing. This is troubling given that the primary recommended treatment in Denmark for infections caused by *Trichophyton* species is terbinafine and that isolates are not routinely susceptibility tested [4,41]. We found an increasing number of patients with terbinafine-resistant isolates compared to previous years (Figure 1). This could both be due to an increase in resistance prevalence and an increase in testing. The number of isolates submitted for susceptibility testing has more than doubled from 2019 to 2020 (19 to 45 isolates). Concomitantly, the percentage of *T. rubrum* isolates with identified mutations or terbinafine resistance has declined (from 14/16 = 88% in 2019 to 17/35 = 49% in 2020), indicating greater awareness among clinicians of the availability of susceptibility testing and more samples being submitted. For the *T. interdigitale/T. mentagrophytes* SC, an actual increase in resistance seems most likely. Since the first detection in 2018, we have found an additional six patients with terbinafine-resistant *T. indotineae,* which has spread rapidly in India and surrounding regions and also been found in an increasing number of European countries [19,20,21,23,42]. This is worrisome, as *T. indotineae* may establish itself in the Danish environment and cause a local epidemic. The clinical manifestation of the infection is often more widespread than other dermatophytoses and as *T. indotineae* is commonly terbinafine-resistant, the risk of epidemic transmission is believed to be higher. Future years will show if this is indeed the case.

The studies has some limitations. In order to allow for prospective monitoring of resistance, we have chosen to present data as a yearly prevalence. This is in contrast to the previous study from 2013–2018, which only included new patients once [6]. Furthermore, we have no clinical or treatment data. To show the MIC variability and the percentage of resistance among received samples, we have included two consecutive isolates from four patients (samples > 60 days apart), as we do not know if this represents reinfection or treatment failure. Finally, we only have full species identification (ITS-based) of susceptibility tested *T. interdigitale/mentagrophytes* SC isolates 2019–2020 and the two resistant isolates 2013–2018 and may have overlooked earlier *T. indotineae* isolates.

## 5. Conclusions

During 2019–2020, we found an increasing number of terbinafine-resistant *Trichophyton* isolates compared to previous years, partly due to a higher awareness and more isolates being submitted. As in previous years, *T. rubrum* was the most prevalent species. Resistant *T. indotineae* was probably introduced around 2018 and it contributes to the terbinafine NWT rate of approx. 60%. Danish clinicians should be aware of the possibility of infections with terbinafine-resistant dermatophytes, especially in wide-spread infections or recalcitrant cases where species identification and susceptibility testing is highly relevant. Finally, SQLE sequencing of all isolates has helped to detect terbinafine resistance in slow growing isolates with mutations associated with more discreet MIC elevations.

## Figures and Tables

**Table 1:** Number of patients and referred *Trichophyton* isolates during 2019–2020.

	Number per Year (in % of Total)		
	2019	2020	2019–2020
Patients	16	43	59
*T. rubrum*	13	35	48 (81.4%)
*T. indotineae*	2	4	6 (10.2%)
*T. interdigitale*	1	3	4 (6.8%)
*T. benhamiae*		1	1 (1.7%)
Isolates	19	44	63
*T. rubrum*	16	35	51 (81.0%)
*T. indotineae*	2	5	7 (11.1%)
*T. interdigitale*	1	3	4 (6.3%)
*T. benhamiae*		1	1 (1.6%)

For three patients with *T. rubrum* and one patient with *T. indotineae*, 2 isolates were included, separated by 70–231 days. All these were terbinafine-resistant and harboured SQLE mutations.

**Table 2:** Antifungal MIC distributions, modal MICs, MIC90s and proportions of *Trichophyton* isolates that are considered NWT for terbinafine, itraconazole and voriconazole.

Species	Drug	MICs (mg/L)													MICs (*n*)	Modal	MIC_90_	Range	%>tECOFF
		≤0.004	0.008	0.016	0.03	0.06	0.125	0.25	0.5	1	2	4	> 4	ND					
*T. rubrum*	TERB	1	3	8	7	1		2	7	4	1	6	3	8	43	ND	ND	≤0.004–>4	55.8
*n* = 51	ITRA			9	8	14	9	2	1			1		7	44	0.06	0.125	≤0.016–4	4.5
	VOR			2	7	20	13	2						7	44	0.06	0.125	≤0.016–0.25	4.5
	ISCO			9	11	17	5							9	42	0.06	0.125	≤0.016–0.125	
	POS			4	11	15	12	2						7	44	0.06	0.125	0.016–0.25	
	OLO	3	5	18	13	3								9	42	0.016	0.03	0.004–0.06	
															0				
*T. indotineae*	TERB										2	3	2		7	4	ND	2–>4	100
*n* = 7	ITRA			4	2	1									7	0.016	ND	≤0.016–0.06	0
	VOR					1	3	2	1						7	0.125	ND	0.06–0.5	0
	ISCO					1	2	2	2						7	ND	ND	0.06–0.5	
	POS		1	3	1	1	1								7	0.016	ND	0.008–0.125	
	OLO		2	3	1									1	6	0.016	ND	0.008–0.03	
*T. interdigitale*	TERB	1	1	2											4	ND	ND	≤0.004–0.016	0
*n* = 4	ITRA			2	1	1									4	ND	ND	≤0.016–0.06	0
	VOR					2	1	1							4	ND	ND	0.06–0.25	0
	ISCO			1	1	1		1							4	ND	ND	≤0.016–0.06	
	POS				3	1									4	ND	ND	0.03–0.06	
	OLO		3	1											4	ND	ND	0.008–0.016	

TERB: terbinafine; ITRA: Itraconazole; VOR: Voriconazole; ISCO: Isavuconazole, POS: Posaconazole; OLO: Olorofim. Dotted red lines show the EUCAST tentative ECOFFs. *T. interdigitale* isolates were considered WT if below the *T. indotineae* tECOFF and having a unimodal distribution. Colour coding used to indicate the most common MICs for terbinafine (the darker the colour, the more isolates with a given MIC). Truncated ranges are marked in grey. Modal MICs are underscored. NWT MICs (MICs above the tECOFF) are marked in bold. The isolate of *T. benhamiae* had the following MICs: TERB and OLO: 0.016 mg/L; POS and ITRA: 0.125 mg/L; VOR and ISCO: 0.25 mg/L.

**Table 3:** Terbinafine MICs (mg/L) in relation to SQLE profiles of *Trichophyton* isolates. The EUCAST tECOFFS are inserted as dotted red lines.

Species	SQLE Profile (Genbank Accession)	MIC (mg/L)													N	% NWT /SQLE
		≤0.004	0.008	0.016	0.03	0.06	0.125	0.25	0.5	1	2	4	>4	NP		
*T. rubrum*	F397L (OM313306/OM313307)								1	2	1	6	1	3	14	60.8
	F397I (OM313305)								1	1					2	
	L393F (OM313304)												2		2	
	L393S (OM313303)							2	5						7	
	Y414C/L438C (OM313302)													1	1	
	F415S (OM313301)													1	1	
	F415V (OM313300)													1	1	
	L437P (OM313299)									1					1	
	H440Y (OM313298)				1										1	
	I479V (OM313297)			1											1	
	WT (OM313296)	1	3	7	6	1								2	20	
*T. indotineae*	F397L (OM313310/ OM313311)										2	2	1		5	63.6
	L393F (OM313308)												1		1	
	F397L/A448T (OM313309)											1			1	
*T. interdigitale*	WT (OM313312)	1	1	2											4	
*T. benhamiae*	WT (OM313313)			1											1	0
*Trichophyton* spp.	Total	2	4	11	7	1		2	7	4	3	9	5	8	63	60.3

NP: Not possible. *T. interdigitale* isolates were considered WT if below the *T. indotineae* tECOFF and having a unimodal distribution. Numbers in red indicate isolates that are NWT, whereas numbers in green indicate WT isolates. Finally, numbers in orange indicate isolates with classification mismatch between SQLE profile and phenotypic resistance.

## Data Availability

Data are only available for research upon reasonable request to Statens Serum Institut and within the framework of the Danish data protection legislation.
