# Correction: Astvad et al. Increasing Terbinafine Resistance in Danish *Trichophyton* Isolates 2019–2020. *J. Fungi* 2022, *8*, 150

**DOI:** 10.3390/jof8080801

**Published:** 2022-07-29

**Authors:** Karen Marie Thyssen Astvad, Rasmus Krøger Hare, Karin Meinike Jørgensen, Ditte Marie Lindhardt Saunte, Philip Kjettinge Thomsen, Maiken Cavling Arendrup

**Affiliations:** 1 Unit of Mycology, Statens Serum Institut, DK-2300 Copenhagen, Denmark; rmj@ssi.dk (R.K.H.); kmj@ssi.dk (K.M.J.); disa@regionsjaelland.dk (D.M.L.S.); maca@ssi.dk (M.C.A.); 2 Department of Dermatology, Zealand University Hospital, DK-4000 Roskilde, Denmark; 3 Department of Clinical Medicine, Faculty of Health Science, University of Copenhagen, DK-2100 Copenhagen, Denmark; 4 Department of Clinical Microbiology, Aalborg University Hospital, DK-9220 Aalborg, Denmark; p.thomsen@rn.dk; 5 Department of Clinical Microbiology, Rigshospitalet, DK-2100 Copenhagen, Denmark

## Error in Citations in the Text

In paragraph four of the discussion in the original article [1], as published, some amino acid alterations from other studies were incorrectly cited.

In the fourth paragraph of the “Discussion” part in the original article, all G408L should be corrected to Q408L (including when in G408L/A448T), and L276A/L419F should be corrected to K276N/L419F.

Additionally, “in our study, also” should be inserted between “F415S and” and “F415V” in the second sentence “In agreement with our findings, prior reports have found that *T. rubrum* isolates with F415S and F415V variants have more retarded growth than WT isolates”.

Finally, a space is missing between the word ”the” and “S443P” in the last sentence “Similarly, theS443P alterations in *T. mentagrophytes* has been found in both resistant and susceptible isolates from India”.

The corrected paragraph is as below:

Moreover, alteration Q408L or Q408L/A448T in *T. mentagrophytes* has been associated with elevated MICs in isolates from Switzerland and India [9,13,18], whereas L335F/A448T and S395P/A448T were associated with discreet MIC elevations in *T. mentagrophytes* isolates from India [13]. In agreement with our findings, prior reports found that *T. rubrum* isolates with F415S and in our study, also F415V variants have more retarded growth than WT isolates [9]. This would indicate that isolates with some mutations may be challenging to susceptibility test and additional information can be obtained through SQLE sequencing. In contrast to the alterations discussed above, some alterations are found in both susceptible and resistant isolates or exclusively in susceptible isolates, suggesting they do not affect the terbinafine susceptibility. This was the case for the I479V alteration found in an isolate with an MIC of 0.016 mg/L and thus identical to the modal MIC of the WT population. Similarly, the S443P alteration in *T. mentagrophytes* has been found in both resistant and susceptible isolates from India [13]; single A448T alternations almost exclusively in susceptible isolates of *T. mentagrophytes* or *T. indotineae* from Germany, Iran, and India [13,21,33,40]; and V444I/A448T and K276N/L419F alterations in *T. mentagrophytes* isolates from Germany or China were not associated with MIC elevations [21,33].

The corrected text appears below. The authors apologize for any inconvenience caused and state that the scientific conclusions are unaffected.
